# Stigma, Partners, Providers and Costs: Potential Barriers to PrEP Uptake among US Women

**DOI:** 10.4172/2155-6113.1000730

**Published:** 2017-09-25

**Authors:** Lakshmi Goparaju, Nathan C Praschan, Lari Warren-Jeanpiere, Laure S Experton, Mary A Young, Seble Kassaye

**Affiliations:** Georgetown University, Washington, D.C., USA

**Keywords:** Pre-exposure prophylaxis, HIV Prevention, Women

## Abstract

**Background:**

Pre-Exposure Prophylaxis (PrEP) use has remained low among US women while significantly increasing among men who have sex with men. Besides lack of awareness, women face several social and structural barriers in gaining access to and using PrEP.

**Methods:**

Four focus group discussions with 20 HIV-negative women who live in the Washington DC metropolitan area.

**Results:**

The women expressed concerns about social and structural barriers to PrEP use. They were afraid that stigma related to using “HIV medicines” could affect PrEP use as well. They are worried that family and friends may question their reasons for taking anti-retrovirals and suspect that they were HIV-positive. They expected hostile reactions from male partners, including accusations of infidelity and introducing mistrust in their relationships. Communicating with health care providers about sexual matters in general and their need for PrEP in particular were identified as further barriers. Women reported that providers rarely ask about risk behaviors related to HIV acquisition; that short visits hinder establishing a trusting relationship to discuss sensitive matters. They were concerned that disclosure of risk behaviors may result in judgmental responses and harsh treatment from providers. Lastly, women were concerned that PrEP costs, including insurance coverage and copays, would keep PrEP out of their reach. While cognizant of the potential barriers, women were unwavering in their determination to find ways to circumvent challenges to PrEP access.

**Conclusion:**

Social and structural barriers may impede women's access to PrEP despite their own reported interest. Continued efforts to reduce HIV stigma, improve patient-provider relationships and ensure affordability of PrEP may increase the likelihood that women will use this important prevention modality.

## Introduction

Pre-Exposure Prophylaxis (PrEP) is the prevention of HIV seroconversion via biomedical intervention. The currently approved regimen for PrEP is daily, oral Truvada (tenofovir-emtricitabine) in combination with safe sex practices (e.g. condom use) among at-risk, sexually active adults [1-3]. PrEP has demonstrated efficacy to prevent HIV transmission in clinical studies worldwide [4,5]. Evidence for effectiveness of this approach in clinical care settings is now starting to emerge [6].

During 2015, 19% of all new HIV infections in the US were diagnosed among women; 86% of them were attributed to heterosexual sex; 76% were among minorities — African American (61%) and Hispanic women (15%) compared to 19% among white women [7]. Despite the potential of greatly reducing risk of HIV infection, PrEP use among women in the United States (US) remains far lower than among men. The number of women who initiated PrEP remained fat around 2,500 per year between January 2012-September 2015; during the same period, numbers of men who initiated PrEP rose about 7 times, from 2,778 in 2012 to 19,344 in 2015 [8]; further, African American and Latino women had much lower rates of PrEP initiation compared to white women despite their higher rates of HIV diagnosis in 2014. The reasons for this disparity remain unclear. Several studies have shown that PrEP awareness is markedly low among women [9-11]. This lack of awareness is further complicated by results from the VOICE and FEM-PrEP studies, which failed to demonstrate significant reductions in HIV seroconversion among women [12-14]. Follow up studies found that efficacy was compromised by poor adherence, burden of daily pill taking, and low risk perception [13]. On the other hand, further research has highlighted the need for high levels of adherence among women to achieve adequate drug levels in the female genital tract tissue [15] providing additional biologic basis for the lack of efficacy that was reported in the VOICE and Fem-PrEP studies. To help address the barriers to adherence, an Adherence Strengthening Program was put in place for participants of the VOICE trial; while it made minimal, if any, differences in adherence, a qualitative review of this program subsequently supported the use of integrative adherence support packages with a special emphasis on understanding the lived experiences of at-risk women and how those experiences influence adherence [16]. Several studies have identified multiple concerns from potential users of PrEP, including side effects, costs and logistics [10,11,17-19]. The multi-city Auerbach et al. study found that while HIV-negative women were interested in PrEP, they identified mistrust of medical professionals and social stigma as two of the most important barriers to PrEP uptake [11].

Women's decisions to take PrEP and their ability to adhere to taking it depend on their broader as well as particular social contexts. Social and structural issues remain some of the most important, albeit poorly understood, factors to women in deciding to take PrEP. Young and McDaid [20] argued that how individuals “view, chose, and maintain the use of particular HIV prevention methods,” in this case PrEP, are “significantly influenced by social, cultural and structural factors.” The authors list the key factors as social stigma, mistrust of medical settings, power imbalance in heterosexual relationships, mistrust of medical settings and structural constraints such as financial barriers and access. Roberts and Mathews reiterated the importance of structural interventions along with biomedical and behavioral interventions for HIV prevention [21]. A study by Cowan and Delaney-Moretlwe focusing on female sex workers, found that structural issues such as stigma from medical professionals, poor social support, and fear of authorities and clients were major barriers to uptake, in addition to risk perception [22]. Ware et al. found that social factors such as supportive relationships with family and sexual partners can improve PrEP uptake and adherence [23]; these findings are similar to those of studies on antiretroviral adherence for HIV-positive individuals [24].

The goal of this paper is to contribute to the understanding of the social and structural barriers facing women, specifically African American and Latina women, living in the Washington D.C metropolitan region with high HIV prevalence, as they endeavor to remain HIV-negative. Findings presented in this paper are part of a qualitative study on women's knowledge, attitudes and behavior about PrEP and Treatment as Prevention, conducted among participants of the Women's Interagency HIV Study (WIHS) in Washington, DC. The WIHS is a long-term, multi-center prospective study of HIV-positive and high-risk HIV-negative women in the United States [25]. Participants of the study visit the site semi-annually for interviewer-administered surveys, physical exams and collection of biological specimens.

## Methods

We used focus group discussions method for this study as it can provide insights into complicated topics. We conducted four focus groups with HIV-negative women during February-May, 2014. At the time of this study, there were 91 active HIV-negative women in the DC WIHS; letters were sent to them informing about the study and inviting their participation. An announcement was also placed in the DC WIHS newsletter. Women who volunteered were scheduled in the order in which their calls were received and based on their availability on the days when focus groups were arranged. Participants signed the informed consent form approved by the Georgetown University IRB. Each participant received $40 cash, transportation assistance and refreshments. Focus groups were conducted in a private conference room at the study site; each one lasted 1.5-2 hours.

### Participants

A total of 20 HIV-negative women participated in the focus group discussions. Their ages ranged from 31 to 61 years and the median age was 50. Of the 20 participants, 16 were African American, 2 were Latina and 2 identified as “other.” The participants' socio demographic data are included in Table 1. The majority (65%) of HIV-negative participants reported having at least one male sex partner in the previous 6 months and only 31% of them used condoms. As participants in the larger WIHS study, the women undergo HIV test during their WIHS semi-annual visit; all tested HIV-negative within 6 months before the focus groups. All participants had close family members or friends that were infected with HIV. In presenting their views in this paper, all participants were given pseudonyms to ensure confidentiality.

After ascertainment of prior knowledge of PrEP, a script based on CDC guidance documents was read to the participants describing what is PrEP, FDA's approval of PrEP and the different aspects of the comprehensive PrEP package: getting a prescription from a provider, taking the pill every day consistently along with using condoms and visiting a provider for HIV testing, blood work and prescription every three months [26,27]. This was followed by discussion to understand women's opinions and concerns about PrEP. The first author moderated all the focus group discussions.

### Data analysis

All the focus group discussions were digitally recorded and later transcribed verbatim by two of the co-authors (LE and NP). They coded the data to facilitate inter-coder reliability and the first author reviewed the coding. NVivo 10 qualitative analysis software was used for coding and analysis. Codes were developed based on the domains of potential barriers as discussed by the women; they were further refined and agreed upon following discussions among the investigators. The study team iteratively discussed the data: after each focus group, and during and after transcribing and coding. We listened to the recordings several times and discussed emerging themes and patterns.

Key topics discussed in the focus groups and presented in this paper include social support or lack thereof for women's PrEP uptake from their immediate social networks such as partners, families, and friends; nature of their past or potential communications with health care providers (HCP) about sexual matters, HIV and PrEP; HIV-related stigma. In discussing these topics women pointed out the social barriers that they may be facing in deciding to take and gain access to PrEP. They also pointed out the potential structural barriers including insurance restrictions and financial costs associated with PrEP that they cannot afford.

## Results

Key findings from this study are as follows: (1) partners, family and friends may have hostile reactions towards women who take PrEP; (2) difficulties in discussing risk behaviors with healthcare providers may hinder the women from seeking PrEP; (3) HIV-related stigma may be extended to taking the PrEP medication and negatively influence people's opinions and actions; (4) medical insurance, lack thereof or its restrictions and high costs of PrEP may impede access. However, enthusiasm of HIV-negative women to use PrEP was not diminished, even in the face of all these challenges. These findings and other concerns raised by the women are presented and discussed in the following sections.

### Social support

Social support from one's social networks plays a crucial role in determining to take and maintain adherence of PrEP. Women discussed a hypothetical situation of how their immediate social networks such as male partners, families and friends would react if they were to find out that the women were taking PrEP and how they would respond to those reactions.

### Male partners' reactions

Perceived reactions of male partners to their female partner's use of PrEP could play an important role in women's decision making. The women thought that their partners would be upset to learn that they were taking PrEP as that would indicate mistrust of partners or infidelity on part of the women.

JADA, age 44: You know if I tell my husband I'm taking this medication he would ask me all these different questions and “Why are you doing it [grunts]? [Instead] What about encouraging me and saying — where is your approbation? Let me see.” Oh my God!

LAURA, age 45: My husband is machismo and he's like, “Why are you doing this?” Just like — he was like, “For what reason? Are you cheating? Are you doing this?”

Similar to other identified barriers to PrEP use, HIV-negative women reaffirmed their willingness to use PrEP inspite of the difficulties it might introduce into their relationships. However, their specific reactions to such perceived indignation from their partners ran the gamut, from complete indifference…

CYNTHIA, age 57: I would say, “I'm taking this [hits the table with her hand]. This is why I'm taking this [hits the table again]. This is how I'm taking it [hits the table again]. I'm taking it to keep myself healthy. And I don't give a fuck of what you think about it!”

ANNA, age 55: It doesn't matter. He doesn't have a choice. I'm gonna protect me.

…to keeping their potential PrEP use a secret…

BRITTANY, age 42: Just like I would — I say I come here [WIHS research visit] for my physical and I'm taking my HIV test. “What, you got something?” And it was always a problem! So I wouldn't tell. Private. Private me. I wouldn't tell.

JADA, age 44: I'd tell him it's for my liver.

…to bargaining with their partner by encouraging them to start taking PrEP as well.

SAKINA, age 53: Well I would have my partner to take it…you know. I would talk to my partner to take it too…I mean, if we together, we gonna do things together.

Ultimately, although the women would manage their partners' reactions in different ways, they saw their relationships as a challenge to overcome but not as deal-breaker for taking PrEP.

### Women's reactions to their male partners taking PrEP

We also asked the women how they would react if they came to know that their male partners were taking PrEP. Their reactions were just as varied as their perceptions of their partners' reactions to their own hypothetical use; some would get angry wondering why the men need to use it invoking issues of cheating and mistrust, while others would feel relieved that their partners were playing safe. Some reported that they would be happy for their partners, yet would feel some distrust.

CYNTHIA, age 57: You know what, a part of me wish — now that you mentioned it, would think, “Well, is he doin' somethin'?” Is that why he need to take it? …But at the same time, I might have a little problem with it but I'm like, “Okay well, I'm glad he's taking it.”

LAURA, age 45: Because I would be — I'm glad he's taken it but like in the back of mind, like she's sayin' [referring to what another participant said], “What is he doing? For what — what is the purpose? Why is he--what's his reasons of doing it?” Just like he would think the same thing about me.

Other participants indicated a preference for PrEP in the face of issues of mistrust, saying that if their partners are going to cheat, then it is better that they are safe about it and in turn protect them, their primary female partners.

SAKINA, age 53: It could be because of he — what he done out there, you know. He wants to be safe to — to not hurt me.

KEISHA, age 57: I would think him being safe, regardless. I would trust him more.

Some women did not care whether or not their partners take PrEP; others were enthusiastic about the possibility of taking it together to take charge of their own health.

JADA, age 44: I would love it!

BRITTANY, age 42: I mean it would be nice if my partner doing it too! ‘Cause I don't know where you go when you walk out the door but, I mean, you got to protect yourself as well.

### Reactions of Families and Friends

Most women reported that their families and friends would be uncomfortable with them taking PrEP because of its association with HIV.

FELICIA, age 44: [They would say] “Oh, what do you have? You have somethin' that I should be concerned with? You can't come over my house no more.” 'Cause there is still people who — who are afraid or cautious about HIV-positive people…and they still separate themselves, thinkin' if you touch 'em you can get it and things like that.

SABRINA, age 49: You know, if you go your family members house with a lot of medication, [they would say] “What you takin' all this medicine for? What you take — what you got? What you got?”

However, some indicated that their social networks would be supportive of their decision to use PrEP, despite the HIV stigma.

CYNTHIA, age 57: They would be so happy to know that I'm taking this initiative to make sure that I'm safe. ‘Course my mother's religious. First thing she'd say is, “If you didn't do it at all,” but I'm doing it. You know. I'm having sex so, they would be happy. And I don't have a problem telling my man or anybody else about it, at all.

MEGAN, age 31: Shoot, they should be happy.

The women who reported that their families and friends would have a negative reaction to their taking PrEP also indicated that they would simply not tell them.

LAURA, age 45: It's none of their business.

BRITTANY, age 42: Like if I was taking it — if I was taking it now, I wouldn't tell — I wouldn't even — my close best friend, I wouldn't even tell because once you have someone negative that you, you know, family members, friends, whatever, certain things you — you tell a little bit, certain things you — and then if you know that they gonna be judgmental…you don't do it. You don't do it. Because your conversation, that's all it's gonna be all day, the next day and so — I wouldn't do it [tell]. I wouldn't do it. I would just keep it to myself and just do what I got to do.

However, not one participant reported that hostile reactions of their families and friends would adversely affect their decisions to use PrEP. Rather, the women in this study voiced every intention to use PrEP and while cognizant of potential and perceived barriers, none were perceived to be insurmountable.

### Difficulties in patient-provider communications

Another barrier the HIV-negative women identified focused on difficulties discussing risk behaviors — theirs or their partners' — with health care providers, which is an important step in obtaining PrEP. Some HIV-negative women detailed humiliating experiences with providers.

FELICIA, age 44: I had one experience when I was in college and I talked to one of the health care providers and I said I was concerned because chlamydia was goin' around on the campus and I didn't wanna get it. And how can I protect myself and, you know, I think I might be exposed or what do I do. And the doctor looked at me like, “[makes noise indicating surprise] Really?” Like almost like, you know, made me feel like, “OK, I'm a horrible person ‘cause I'm talking about all these things.”

[…] So that made me shut down with wanting to talk to health care providers.

SABRINA, age 49: …I never, uh, mention anything to none of my doctors unless it's somethin' seriously is wrong with me or somethin' internally is goin' on with me. Because I feel like if you go to a doctor's office, you know and you reach out to them, some may be carin' and lovin' and some just don't care, you know.

The participants explained that good rapport with a healthcare provider is essential for them to freely discuss their risky sexual behaviors. The women who said they would be open to discussing their sexual behaviors did so because they liked and trusted their doctors and had been seeing them over a long period of time. Women felt more comfortable to talk to their therapists about their risky behaviors rather than to their doctors.

GISELLE, age 34: If I had multiple partners, I wouldn't discuss that with my doctor, saying, “I'm having sex with all these men.” ‘Cause there's really nothing they can do for that. I would have to make the decision (to change behavior)…may be a psychologist. You can talk to a therapist about, I think, your sexual behavior. But I don't think I would discuss with my doctor about risk ‘cause I – they would tell you to stop or use protection, I'm assuming.

Others reported that while they are generally uncomfortable discussing their risky sexual behaviors with providers, they would be more open to it in the context of PrEP. Self-assessment of risk was a key factor in deciding to discuss sexual practices with providers.

CYNTHIA, age 57: I would only tell my doctor if it was necessary. If I wanted that pill and felt that I needed it, I would tell him. But, in general, no I don't tell — talk to my doctor about things like that.

Women also pointed out that their interactions with healthcare providers are short, and they often have no time to feel comfortable to disclose their risk.

MEGAN, age 31: ‘Cause that's how they do your little checkup. Rush you in and rush you out. So, it's like you don't even have time to talk to them.

SABRINA, age 49: (Doctors rush through their appointments) without even tellin' you how your tests came out.

Social stigma around discussion of sexual matters, particularly socially stigmatized behaviors such as risky sexual behaviors, influences both the providers and the patients. This is further complicated by the shorter time slots provided for the interaction.

### Impact of HIV/AIDS stigma on PrEP uptake

HIV related stigma is apparently extended to PrEP use. Perceived opinions of social networks as well as interactions with providers are influenced by HIV stigma — sometimes internalized and at other times externalized. The women indicated that they have observed HIV/AIDS stigma in their daily lives. HIV/AIDS is often discussed in their social circles, but rarely in a positive or supportive manner. For example:

SABRINA, age 49: So, my family is basically — I got like 50- 60% [of our conversations], that's on a personal level. And the other 40% is always on a negative level. Somebody gonna say, “Oh, what you got? AIDS or somethin' ? You takin' all this damn shit. You got AIDS or somethin'?”

While Sabrina discussed externalized stigma, Brittany spoke about internalized stigma.

BRITTANY, age 42: It would be because people would think, of the break, something is wrong with you. Just like being in this program [research study], my sister always thought I was HIV-positive.

The women expressed frustration about lack of genuine discussions in their social circles.

JULIANA, age 61: I took that book [a children's book about HIV] and I was going to give it to my granddaughter at the time. And she was about six. And my daughter did not, under any circumstances, want me to give her that book. She did not want her [child] to know anything about HIV and still I doubt if she's ever talked to her [child] about it. So I think people are afraid to have that conversation here. And I think that, people just don't want it to be a reality, even though it is.

Some reported that HIV stigma impacts their conversations even with providers. HIV disease carries stigma; their being involved in risk behaviors or being a partner to someone who is involved in risk behaviors also invokes social stigma attached to such behaviors. Thus, internalized and externalized stigma related to HIV is sometimes further complicated with social stigma attached to socially-not-accepted sexual or drug use behaviors.

GISELLE, age 34: These are so emotional, these questions. No — I don't — there's a limited population who will speak freely about that, I think, to their doctor. To have that conversation. To say, you know, “I'm worried because I think my man…” Tat — that takes a lot.

MODERATOR: Why?

GISELLE, age 34: I just — the stigma…judgment. Humiliation, too…Tat's a very difficult conversation, I would assume, to have with a physician or anyone.

The women expressed concern that people taking PrEP may face stigma for their use of HIV medications, regardless of their serostatus.

FELICIA, age 44: I think the whole stigma, the HIV stigma, and this is HIV medication that you're taking. I think that could be a big stigma to it.

However, women reacted to the challenge of stigma in the same way that they reacted to other barriers to use PrEP: it would be difficult to deal with, but it would not deter them from taking PrEP. Some were even dismayed that anyone would let stigma keep them from preventing seroconversion:

ANNA, age 55: I cannot see, as dangerous as HIV and AIDS is; as dangerous as it is, I cannot see a negative stigma behind this medication. This is crazy. This is a possibility that you will never in your life get it and somebody's gonna think negative. They — no, that's crazy.

### PrEP guidelines and financial costs

Even if the women could cross the barriers of their social networks' objections, were able to effectively communicate with their providers about their need for PrEP and resolve their own and societal stigma, that did not necessarily quell concerns for some. Women indicated that they would be concerned about the PrEP guidelines issued by larger medical organizations such as CDC which may not place them in “high risk” category. Additionally, they feared that medical insurance and its coverage restrictions or complete lack of insurance, may keep them from obtaining PrEP.

CYNTHIA, age 57: Ten, I'm thinking, what you are going to have to go through? …Who is going to be considered high-risk? You just can't go to the doctor and say, um, “Well yeah, I have been having sex” or “I'm — I've been out there using drugs and I'm out there…Can I get it?” Even though your doctor may be okay with this, especially if you had the doctor for a long time, he knows you. But then, whatever the guidelines are to get it and then your insurance to pay it, it's probably — it may be a whole lot to go through.

Women who were involved in sex work expressed concern about age range for PrEP use.

TAWANA, age 37: what's the age range? Because I know, when I first started (sex work) - that pill sound like the best thing ever…What is considered adult? ‘cause I know 13 is adult…having sex…

CDC guidelines on prescribing PrEP remind clinicians that the FDA's PrEP indications are specified for “adults,” but an exact age definition is not provided; and that the completed PrEP trials only included individuals above the age of 18; clinicians are advised that they must weigh the benefits and risks of taking PrEP for individuals under 18 [11,26]. Women expressed concern that this guideline may preclude individuals under the age of 18 years from accessing PrEP.

The price of PrEP or co-pays for those with insurance, could be a significant barrier for many women. We asked the women who they think should be paying for PrEP; whether they would be willing to pay and how much. We asked them to think of their budgets, their own risk or assumed risk, and the importance of PrEP in their life; taking those factors into account, how much would they be willing to pay per month for PrEP. Several of them were willing to pay small sums of money. Some thought of giving up luxuries such as buying shoes, doing hair and nails, so that they could pay for PrEP. Many would be willing to pay $20 a month; a few allowed for costs as high as $100 a month. Others agreed with the principle of cutting back on some luxuries to afford PrEP, but reported that they do not do nails, hair, etc., as they do not have money, and thus have nothing that they can give up to pay for PrEP. Many indicated that they could not afford to pay anything. Some reported that they were homeless and had no money to pay.

JADA, age 44: But…basic of my situation [hits the table with hand], I'm not even — haven't bought no clothes. Honestly y'all. In two years.

CYNTHIA, age 57: I don't really know ‘cause, um…I don't know. I can't answer that. I really don't know. ‘Cause right now my situation and my income and—I'm homeless. I don't even have a place to live. So, I don't really know (how much I can afford to pay) at this moment.

The women felt that health insurance or Medicaid should cover the costs of PrEP. Some others who originally wanted to use PrEP said they would not be able to take it if they have to pay for it.

VANESSA, age 51: If Medicaid or Medicare can't pay for it, I won't be gettin' it.

JADA, age 44: If Medicaid can't pay for it and I can't get an organization to assist me with it [knocks on the table], I'm dead out! And — and — it would really hurt me a lot because I know I need that medication!

Some thought that they would have to change their behaviors as they could not afford to pay.

STEPHANIE, age 49: If it comes between my money and being high-risk, I'm stopping high-risk [laughs]…‘cause I can't afford it [says this to herself].

AMBER, age 51: My money goes on to (pay) my bills. So if it comes to the medicine or stop doing my bad behavior, I'd just stop doing my bad behavior. Simple as that. ‘Cause I don't have the money to waste.

Others argued that given the importance of HIV prevention, PrEP should be provided free of charge.

ANNA, age 55: Bein' as though this is a epidemic, you know, and it's about livin' and dyin' in this society, it should be free just like rubbers.

However, despite being homeless and not having money even for clothes, Cynthia did not give up on the hopes of PrEP and wanted to do her best to pay.

CYNTHIA, age 57: If Medicaid or something didn't pay for it, I would definitely do my best to seek an organization, or some kind of program, that would help me. I just — they don't have to pay the whole thing if they can help me pay it. Ten that wouldn't be a problem.

Women who have jobs were willing to pay slightly higher sums per month, though everyone emphasized the importance of affordability. They thought PrEP as a necessity, so should be paid for, but they hoped that it would be within reach. “If I could afford it,” was a refrain we heard often.

ANNA, age 55, It's sad. But I just hope if people have to pay for it, they will make it where we can afford to pay for it because I would pay for that. I would think that would be worth payin' for. Just like toilet paper or whatever. Some things that you are nec — you know, is necessity in the house. I think that's necessity for me because I'm not with an individual. […] I think I would treat 'em like vitamins. Take 'em as much as I can, as often as I can, when I can afford it.

Women also wondered whether their regular doctors, who were not infectious disease specialists, can prescribe PrEP.

## Discussion

The current study adds to the literature by identifying social and structural barriers that may keep women from trying to access and using PrEP [11,22,23]. The barriers described in this study corroborated those found previously, and included stigma, responses of social circles to PrEP uptake, difficulties in discussing PrEP and related sexual matters with healthcare providers, provision or lack thereof of medical insurance and its restrictions and women's inability to pay for PrEP.

In the focus groups, we did not specifically ask about stigma. Rather, the topic was raised organically throughout our discussions, in all groups; women invoked the issue of stigma when they talked about their social networks, their own attitudes towards PrEP, interactions with providers — they saw it everywhere, despite their own seronegative status. HIV/AIDS stigma or general negative perceptions of HIV and AIDS held by society and manifested in personal interactions, is not a new phenomenon in the epidemic. HIV/AIDS stigma has long been considered a significant burden facing HIV-positive individuals, often preventing them from accessing care [28]. Furthermore, the experience of stigma is associated with internalization of negative social cues among HIV-positive individuals, leading to depression, which is in itself a risk factor of poor adherence to antiretroviral medications [29]. Women taking antiretroviral prophylaxis for the prevention of HIV may also be effected by HIV related stigma — both internalized and externalized — potentially discouraging them from taking PrEP or leading to poor adherence. Per the women in this study, stigma is manifested in the way their friends and families talk about HIV/ AIDS; in the way people with HIV are treated; in the fear of obtaining treatment or prevention. Negative perceptions surrounding HIV may be extended to HIV-negative individuals seeking to reduce their risk of acquiring HIV. As described by Mahajan et al., by seeking prophylaxis, a woman may associate herself with HIV and thus face stigma and self- discriminate by neglecting to talk to her healthcare provider about her risk behaviors; her family, friends and partner(s) may treat her poorly if she starts taking PrEP and she may become labeled and stereotyped as a woman at high-risk of HIV, one whom should be avoided [28]. These stigmatizing events for a woman were all identified by the participants in this study.

Unfortunately, stigma-reduction strategies tend to be low-priority. A greater focus on reducing stigma in the community may be necessary for PrEP to be a viable prevention strategy for women. In sub-Saharan Africa, leveraging social relationships for ART adherence has proven to be highly successful. Based on the Partners PrEP Study, Ware et al. [23] propose that improving peer support and positive role modeling can improve adherence by reducing stigma and increasing social integration. Given the importance of these factors as identified by our participants, a similar social integration program may be beneficial for potential PrEP users in the United States.

The women of this study identified their social networks as a potential barrier for PrEP use. They indicated that their families and friends would mostly have adverse reactions to their taking PrEP. Male partners play an important role in a woman's decision to take PrEP [17]; their opposition could have a negative impact and may lead to women not taking PrEP or forcing them to hide their PrEP use as discussed by the women of this study. The concept of trust in relationships is an inherent challenge around PrEP uptake. Male partners may feel upset by a woman's PrEP uptake, seemingly an indicator of distrust in relationships. This conflict for women interested in prevention is similar to negotiating condom use. How does a woman protect herself while also demonstrating that she trusts her partner, or that she is being faithful? Several women in this study proposed their own solutions, including keeping their use hidden, which is more feasible with PrEP than with condoms; encouraging mutual use of PrEP; and simply standing up to their partners with their need to look out for themselves. Some researchers have identified disclosure and involved partners as important predictors of uptake and adherence to PrEP regimens in Zimbabwe [30]. Some of the women in our study indicated that their families would be supportive. Personal experiences with and knowledge of HIV/AIDS, stronger interpersonal relationships, greater awareness of HIV stigma, knowledge and acceptance of a woman's sexual history — all of these factors may play a role in determining a family member's/ friend's reactions to a woman using PrEP. It remains to be seen whether encouraging partner involvement in PrEP will be beneficial for women in the United States.

Power dynamics in heterosexual relationships are even more important in the case of women who are survivors of intimate partner violence. While the women in our study did not discuss abusive partners specifically, many indicated the reactions of their partners would be aggressive, if not violent. The women who may benefit most from using PrEP are also at the highest risk and may face some of the steepest barriers [31]. The women in our study appear to face many of the barriers as discussed by Braksmajer et al. [31], including partner jealousy and anxiety, difficulties with covert use, and negotiating gender norms. To address these barriers, empowerment programs similar to the EVOLUTION trial described by Brothers et al. [32] may be useful; this group-based, comprehensive program successfully improved adherence to antiretroviral therapy (ART) by empowering women to negotiate gender roles, cultural norms and the power dynamics of their sexual relationships while improving their social support, self-efficacy and self-confidence. Support groups of and for women taking PrEP may be helpful.

Also identified were concerns about a provider's reactions to or a woman's discomfort with discussing risky sexual behaviors. Often, providers do not collect comprehensive sexual history [33,34]. Provider training to overcome discomfort and bias in taking sexual history is important. Use of sexual history tools such as developed by Lanier et al. [35] collect details on specific exposures allowing for an accurate assessment of HIV risk. Discussions with providers about unsafe sex practices represent an excellent opportunity for risk reduction. However, as few as half of encounters of HIV-positive individuals involve meaningful counseling about transmission risk [36]. These findings are reaffirmed by the present study: the women described providers as reluctant to discuss sexual matters with them or as judgmental and negative. Additionally, the quality of a clinical encounter is important not just for risk reduction, but also for PrEP initiation: several studies have demonstrated the importance of a healthcare provider's input in PrEP uptake, especially for African American women [37-39]. For PrEP to be an acceptable tool of prevention, both providers and women must be comfortable discussing risky behaviors; physicians will need to effectively evaluate a woman's risk before making the recommendation for PrEP. Furthermore, a candid relationship with a provider can improve adherence to ART [38]. Unfortunately, providers still represent a significant barrier for women seeking PrEP. In a recent study, fewer than 1 in 5 physicians reported experience with antiretroviral prophylaxis [40]. According to them, barriers to recommending PrEP include risk compensation, financial burdens, and undue side effects; and a small minority raised philosophical concerns about its use as a prevention strategy. Based on these and our study findings, it may be beneficial for education campaigns targeting healthcare providers, particularly in primary care settings with high concentrations of HIV-positive populations, to familiarize them with the barriers facing women interested in PrEP; that could help facilitate the process whereby an interested woman may obtain PrEP.

## Conclusion

The current study findings are important in understanding barriers for women's PrEP use; however, it is limited in scope, diversity, and generalizability. The large majority of our participants were African American. The experiences and social structures in communities of other ethnic and social groups, and those outside the metropolitan DC area, may alter the motivators and barriers for a woman in those communities seeking PrEP. Age range of our participants was 31-61; younger women may approach PrEP differently. Most importantly, the women in this study discussed “potential barriers” to PrEP use rather than actual barriers, as they were not on PrEP. Follow-up studies with women who are taking PrEP may provide greater insights into real-life barriers faced by women, and the ways they circumvented those challenges.

It is clear that women are excited about PrEP. Their willingness to pay for PrEP despite the additional financial burden it would pose on their limited budgets indicates as much, as does their continued enthusiasm throughout all of the focus group discussions. This enthusiasm did not relent even as women named the multitude of obstacles, difficulties, and barriers they face. In light of our findings, in concert with those of others', the following interventions would help to promote women's access and adherence to PrEP: education and involvement of male partners via a public health program in general and aimed at partners of women who are seeking PrEP in particular; improving provider education about risk factors of PrEP and appropriate sexual-history taking to promote comfort in discussion; and HIV related stigma reduction programs at the larger level. This would help reduce some of the new infections among women.

## Figures and Tables

**Table 1:** Demographic characteristics of study participants.

Variable	HIV (N=20)
***Race***	
Black/African-American	16 (80%)
Latina/Hispanic	2 (10%)
Other	2 (10%)
***Age***	
Median	50.0
Average	48.8
***Education***	
No schooling	1 (5%)
Grade 7-11	4 (20%)
Complete high school	5 (25%)
Some college	5 (25%)
Complete college	1 (5%)
Graduate school	4 (20%)
***Employment status***	
Unemployed	11 (55%)
Employed	9 (45%)
***Average Household Income per Year***	
$6000 or less	2 (10%)
$6000-$12000	7 (35%)
$12001-$18000	2 (10%)
$18001-$24000	2 (10%)
$24001-$30000	0 (0%)
$30001-$36000	0 (0%)
$36001-$75000	6 (30%)
>$75000	1 (5%)
***Housing Status***	
Own house/apartment	16 (80%)
Someone else house/apart	3 (15%)
Parents house	1 (5%)
***Relationship Status***	
Currently married	7 (35%)
Not married, but living with partner	2 (10%)
Never married	6 (30%)
Divorced and other	5 (25%)
***Number of Male Sex Partners in last 6 months***	
0	7 (35%)
1	10 (50%)
2	2 (10%)
3	1 (5%)
***Use of Male Condoms among women who had male partners in last 6 months***	
Yes	4 (31%)
No	9 (69%)
***Health Care Provider***	
Yes	18 (90%)
No	2 (10%)
